# Editorial: Neural Mechanisms of Perceptual Categorization as Precursors to Speech Perception

**DOI:** 10.3389/fnins.2017.00069

**Published:** 2017-02-14

**Authors:** Einat Liebenthal, Lynne E. Bernstein

**Affiliations:** ^1^Department of Psychiatry, Brigham and Women's Hospital, Harvard Medical SchoolBoston, MA, USA; ^2^Department of Speech and Hearing Science, George Washington UniversityWashington, DC, USA

**Keywords:** speech perception, phonemic perception, categorization, category learning, auditory processing, audiovisual processing, neural mechanism, neuroimaging

This research topic describes recent advances in understanding the brain functional organization for sensory categorization along with its implications for speech perception. Among the 14 papers, one theme is how neural representations of auditory and visual input are transformed across different scales of neural organization to enable speech perception, and another is the neural mechanisms of category learning.

In the first theme, several animal and human studies delve into the complex hierarchical organization of auditory ventral pathways for speech perception. Prior work has established an important role for the auditory ventral stream in complex sound categorization (Rauschecker and Scott, 2009; Romanski and Averbeck, 2009). In humans, a preference has convincingly been demonstrated for phonemic over non-phonemic sounds in non-primary auditory fields in the middle of the ventrolateral superior temporal cortex (mSTG/S) (Liebenthal et al., 2005; Leaver and Rauschecker, 2010). The present papers contribute novel insights about the function of dorsal areas in and near the auditory core, the functional specificity of the mSTG/S, and the role of non-auditory areas, for phonemic perception. Collectively, they suggest that multiple stages of abstraction from the original form of speech occur in low-level sensory cortices. In the mSTG/S, the neural representations are highly specific to phonemic categories.

Tsunada and Cohen's review of research in the monkey suggests that single neurons in the auditory core encode categories for simple sounds (e.g., direction of spectral changes), whereas neurons in the auditory belt encode more complex categories (including speech phonemes) based on input from the entire population of core neurons. At the cellular level, they report the intriguing finding that different classes of neurons within the auditory belt may have different sensitivity to category information: The more common pyramidal neurons encode auditory categories with less sensitivity than the less common interneurons (Tsunada et al., 2012). Astikainen et al. also show that in anesthetized rats' primary auditory cortex, neurons automatically encode structural patterns (order of syllable repetition) from a fast paced speech stream and generalize to novel patterns.

Based on intracranial high-gamma electrophysiological recordings in subjects with intractable epilepsy, Steinschneider et al. propose that within 200 ms, activity in the human primary and non-primary auditory cortices reflects non-categorical spectrotemporal sound attributes. Only later, activity in non-primary auditory areas receiving modulatory input from higher-order, lexico-semantic associative cortex represents phoneme categories.

Using multivariate pattern analysis (MVPA) of functional magnetic resonance imaging (fMRI) data, Joanisse and Desouza suggest that primary and non-primary areas in the human auditory cortex encode the direction of frequency modulations of complex non-speech sounds. Using an fMRI adaptation paradigm, Humphries et al. show that a relatively large area in the dorsolateral superior temporal cortex is sensitive to complex acoustic patterns in phonemic and non-phonemic sounds, whereas a small portion of the ventrolateral superior temporal cortex responds specifically to phonemic sounds, with relatively little overlap between the areas. In addition, an area of the medial superior temporal plane shows a preference for non-phonemic sounds. The results support a multi-stage hierarchical stream for speech perception extending from the superior temporal plane to the superior temporal sulcus.

Liebenthal et al. present a large meta-analysis of neuroimaging studies of the left superior temporal cortex, and find a strong preference for speech perception over other language functions in the mSTG/S. This area preferred linguistic over non-linguistic input and auditory over visual processing, prompting the suggestion that a high functional specificity of the left mSTS for auditory speech may be an important means by which the human brain achieves its exquisite affinity and efficiency for native speech perception.

Bernstein and Liebenthal's review of visual speech proposes a neural model of speech perception according to which visual aspects of speech are represented hierarchically in ascending visual pathways, with a functional organization similar to that of auditory pathways. Central to the model is the proposal that a visual area in the left posterior temporal cortex represents visual phoneme categories.

The second theme concerns how altered experience and training regimes affect perceptual categorization and neural processes. Current understanding of the normative organization of speech categories is based mostly on experiments with adults who have experienced normal language acquisition and who listen in their native language. Experiments that use natural or artificial factors that perturb and change the system help to further define the organization and mechanisms of categorization.

Heald et al. report on pitch categorization. They suggest that individuals vary in the extent to which they rely on an internal systematic tone organization. Absolute pitch (AP) possessors may be more analogous to speech perceivers than non-AP musical experts, and musical novices are expected to be least able to categorize tones based on internal organization. All three types of participants were influenced by the structure of the stimulus set and possessed useful prior pitch knowledge. Increased expertise was associated with greater influence of internal category structure.

Myers reviews the literature on normative category processing and suggests that second-language learning involves remapping the native language perceptual space to the perceptual space of the second language. Training studies typically use explicit category training, and Myers points to a wide network of frontal and temporal areas that is recruited as a result of such training. She suggests that learned sensitivity to categories is first observable in the frontal lobe and with greater expertise is observable in temporal areas. This shift is consistent with the reverse hierarchy theory (Ahissar and Hochstein, 1997; Ahissar et al., 2008) and with frontal-to-temporal feedback as a mechanism that assists in warping category representations for the second language.

Callan et al. report an fMRI study comparing English and Japanese speakers listening to native and accented English /r/-/l/. The accented English of Japanese natives is difficult for native English speakers and the English /r/-/l/ is a difficult distinction for native Japanese speakers. In their results, temporal cortex areas are not significantly modulated by expertise. Instead, more difficult distinctions recruit the right cerebellum and left premotor cortex (PMC) in both groups. Second language listening additionally recruits the right PMC and left cerebellum.

Ley et al. discuss the value of MVPA for revealing high plasticity of sound representation in auditory temporal areas as a function of experience and learning. They suggest that sensory plasticity and attention processes interact to mediate category learning. They review findings within predictive coding models of perceptual learning and categorization that support a hierarchical architecture in which variation in sensory information confronts top-down signals that update bottom-up representations.

Scharinger et al. discuss the role of auditory attention in realistic listening conditions, when perception needs to adapt to dynamic degradation of certain stimulus cues. They use multimodal neuroimaging of oscillatory activity in the alpha band to study auditory categorization and highlight the role of posterior auditory areas and the inferior parietal cortex for optimal utilization of informative stimulus cues and inhibition of uninformative cues.

Lim et al. approach speech categorization through the perspective of cognitive neuroscience models that attempt to account for multiple learning systems and corresponding neural structures. These authors frame questions about the relationships between frontal and temporal cortices during learning within larger networks that include the basal ganglia. They discuss different types of feedback and task structure that may eventuate in different types of learning, declarative vs. procedural (Ashby et al., 1998). Category training tasks that encourage trainees to engage in *explicit* attempts to discover categorization rules or structure (declarative learning) result in limited generalization for speech categories, which are inherently multidimensional and incommensurate. Speech category learning appears to require procedural learning that involves bottom-up integration of stimulus features and dopaminergic reward signals.

Bernstein et al. behavioral study demonstrates an advantage to training with audiovisual speech in order to obtain improvements in the auditory perception of vocoded speech. Training used a paired associates task for which participants attempted to learn the associations between disyllabic non-sense words and non-sense pictures. Feedback was for association choices and not the phonemic content of the training stimuli. The audiovisual advantage is interpreted within a multisensory extension of reverse hierarchy theory (Ahissar and Hochstein, 1997; Ahissar et al., 2008): Higher-level visual speech representations during audiovisual training may guide the top-down search for to-be-learned acoustic phonetic features. The training task may also promote procedural learning of the type described by Lim et al.

Future research should build on these insights to advance understanding of the neural basis of speech perception and learning.

## Author contributions

EL and LB contributed equally to the conceptualization and editing of the research topic. EL and LB wrote the editorial together.

## Funding

The work was supported by NIH R01 DC006287 and NIH R21 DC012634.

### Conflict of interest statement

The authors declare that the research was conducted in the absence of any commercial or financial relationships that could be construed as a potential conflict of interest.
